# Severely restricting energy intake for 24 h does not affect markers of bone metabolism at rest or in response to re-feeding

**DOI:** 10.1007/s00394-020-02186-4

**Published:** 2020-02-03

**Authors:** David J. Clayton, Lewis J. James, Craig Sale, Iain Templeman, James A. Betts, Ian Varley

**Affiliations:** 1 National Centre for Sport and Exercise Medicine, School of Sport, Exercise and Health Sciences, Loughborough University, Loughborough, Leicestershire, LE11 3TU UK; 2 School of Science and Technology, Nottingham Trent University, Nottingham, NG11 8NS UK; 3 Department for Health, University of Bath, Bath, BA2 7AY UK

**Keywords:** Intermittent energy restriction, Intermittent fasting, Bone, Bone metabolism, Weight management

## Abstract

**Purpose:**

Intermittent energy restriction commonly refers to ad libitum energy intake punctuated with 24 h periods of severe energy restriction. This can improve markers of metabolic health but the effects on bone metabolism are unknown. This study assessed how 24 h severe energy restriction and subsequent refeeding affected markers of bone turnover.

**Methods:**

In a randomised order, 16 lean men and women completed 2, 48 h trials over 3 days. On day 1, participants consumed a 24 h diet providing 100% [EB: 9.27 (1.43) MJ] or 25% [ER: 2.33 (0.34) MJ] of estimated energy requirements. On day 2, participants consumed a standardised breakfast (08:00), followed by an ad libitum lunch (12:00) and dinner (19:30). Participants then fasted overnight, returning on day 3. Plasma concentrations of C-terminal telopeptide of type I collagen (CTX), procollagen type 1 N-terminal propeptide (P1NP) and parathyroid hormone (PTH) were assessed as indices of bone metabolism after an overnight fast on days 1–3, and for 4 h after breakfast on day 2.

**Results:**

There were no differences between trials in fasting concentrations of CTX, P1NP or PTH on days 1–3 (*P* > 0.512). During both trials, consuming breakfast reduced CTX between 1 and 4 h (*P* < 0.001) and PTH between 1 and 2 h (*P* < 0.05), but did not affect P1NP (*P* = 0.773) Postprandial responses for CTX (*P* = 0.157), P1NP (*P* = 0.148) and PTH (*P* = 0.575) were not different between trials. Ad libitum energy intake on day 2 was greater on ER [12.62 (2.46) MJ] than EB [11.91 (2.49) MJ].

**Conclusions:**

Twenty-four hour severe energy restriction does not affect markers of bone metabolism.

**Electronic supplementary material:**

The online version of this article (10.1007/s00394-020-02186-4) contains supplementary material, which is available to authorized users.

## Introduction

Net energy balance over time will dictate changes in body mass, with a chronic positive energy balance increasing both tissue mass and the risk of several chronic diseases [1]. For most people, weight gain tends to occur during early to middle adulthood (18–49 years of age), with modest yearly weight increments eventually leading to a substantial excess of adipose tissue and increased risk of obesity-related chronic diseases [2]. This indicates strategies to help facilitate attaining and/or sustaining a healthy proportion of body fat are required.

Imposing a daily limit on energy intake, such as reducing habitual energy intake by 20–50%, is a commonly used method of achieving a healthy weight [3]. This method does not, however, appear to be effective for long-term maintenance of a healthy weight in most people [4], with the requirement for daily adherence a likely barrier to success [5]. One alternative method is intermittent energy restriction, which nominally involves alternating between periods of severely reduced energy intake (by ~ 75%) with periods of adequate or ad libitum energy intake [6]. Intermittent energy restriction can achieve similar weight loss and reduction in risk markers of obesity-related diseases compared to continuous energy restriction [7], suggesting this may be a viable alternative weight management strategy.

It is unknown whether severe energy restriction and the resultant acute perturbation of energy balance influences bone remodelling (i.e. osteoclastic bone resorption and osteoblastic bone formation). Bone mineral density is dictated by the balance between bone formation and resorption that occurs over time, which is a reflection of the physiological and mechanical environment [8]. Energy restriction is a risk factor for stress fracture incidence [9, 10], bone metabolism is markedly disrupted by 3–5 days of energy restriction (i.e. 10–30 kcal kg LBM^−1^ day^−1^), as evidenced by reductions in plasma markers of bone formation but without a corresponding decrease in bone resorption markers [11, 12]. Whilst these studies clearly demonstrate the potential for sustained moderate–severe energy restriction to impair bone health, typically, intermittent energy restriction involves shorter periods (1–2 days) of limited energy intake (~ 10–15 kcal kg LBM^−1^ day^−1^) but interspersed with refeeding periods [6]. Such cyclic energy restriction and replacement over serial days, therefore, represent a different model for which the daily kinetics of bone metabolism have never been assessed. One recent study has reported that the accumulated effect of 6 months of alternating daily between 25 and 125% of energy requirements did not alter post-absorptive markers of bone resorption (CTX, osteocalcin) or formation (bone alkaline phosphatase) at follow-up relative to standard continuous energy restriction or control [13]. However, this study employed a self-selected diet in the final 3 months of the intervention, with food diaries revealing only ~ 500 kcal difference between ‘fed’ and ‘fasting’ days in the alternate-day fasting condition. As such, the acute day-to-day variance in bone metabolism between the fasted days and subsequent refeeding days remains unknown.

Feeding is an essential stimulus for bone tissue accretion, as it causes an immediate postprandial reduction in markers of bone resorption and increases in markers of bone formation [14-16]. The majority of previous studies have assessed markers of bone metabolism in the fasted state, but the magnitude and duration of the response in bone metabolic markers after food intake may have a critical impact on bone remodelling. Recent studies have observed that the effects of short-term (1–2 days) severe energy restriction (consuming 10–15 kcal kg LBM^−1^ day^−1^) are not readily apparent in the fasted-state, but become pronounced upon refeeding. When comparing severe energy restriction diets to energy balanced maintenance control diets, previous studies have shown alterations in postprandial glucose, insulin, fatty acids, glucagon-like peptide-1, peptide-P and ghrelin after 24–48 h of severe energy restriction [17-20], with several of these hormones/substrates also postulated to influence bone remodelling [15].

To understand the efficacy and possible bone health effects of intermittent energy restriction diets, it is important to determine how a short period of severe energy restriction affects markers of bone remodelling upon refeeding. This will elucidate how intermittent energy restriction might affect bone health. Accordingly, this experiment investigated how markers of bone turnover respond temporally to an acute episode of severe energy restriction and refeeding, compared to an energy balanced control trial. This study was performed in young, lean individuals to investigate the effect of this eating pattern as a means to prevent weight gain.

## Methods

### Participants

This is a secondary analysis of a study that compared 24 h diets of 25% (ER) and 100% (EB) of estimated energy requirements (EER) on appetite regulation and energy intake [17]. This trial is registered at https://www.clinicaltrials.gov.uk as NCT02696772. Eighteen men (*n* = 10) and women (*n* = 8) were recruited and provided written informed consent to take part in the original study. Two men were removed from this analysis due to issues with obtaining blood samples during 1 trial (Table 1). Participants were healthy, weight stable (self-reported) and recreationally active (3–10 h week^−1^).

**Table 1:** Participant baseline characteristics

	Male (*n* = 8)	Female (*n* = 8)	Combined (*n* = 16)
Age (years)	23 (2)	21 (2)	22 (2)
Weight (kg)	75.6 (6.6)	63.8 (8.6)	69.7 (9.6)
Height (m)	1.80 (0.04)	1.61 (0.05)	1.70 (0.11)
BMI (kg m^−2^)	23.4 (2.2)	24.5 (2.3)	24.0 (2.2)
Body fat (%)	14.3 (3.4)	27.3 (5.0)	20.8 (7.9)

Values are mean (1SD)

### Study design

Height, mass and body fat percentage were determined during a 1-day preliminary trial. Participants then completed two 3-day experimental trials administered in a randomised, crossover, and counterbalanced order. Trials were separated by at least 14 days for men and by exactly one menstrual cycle for women, who were tested during the post-menstruation follicular phase (5–12 days after the onset of menstruation) [21].

### Protocol

During the 2 days immediately preceding the first experimental trial, participants recorded dietary intake and habitual physical activity, which was subsequently repeated in the 2 days immediately preceding the second experimental trial. Alcohol consumption and moderate/vigorous exercise were not permitted during this time or during the experimental trials. For each trial, participants arrived at the laboratory at 07:30 on three consecutive mornings having fasted at least 10 h, and post-void nude body mass was measured.

On day 1, a blood sample was collected via venepuncture (− 24 h), after which (08:30) participants left the laboratory with a 24 h diet providing either 25% or 100% of EER (resting metabolic rate estimated from Mifflin et al*.* [22] multiplied by 1.4) and instructions on when to consume each item. During EB, 100% of EER [9.27 (1.43) MJ] was distributed into four meals: 20% (of total food energy on EB) at 08:00 (cereal, milk and orange juice), 30% at 12:00 (white bread, chicken, mayonnaise, salad and cookies), 10% at 16:00 (yogurt and cereal bar), and 40% at 19:00 (pasta, chicken, Bolognese sauce, olive oil and cookies). During ER, 25% of EER [2.33 (0.34) MJ] was divided between two meals: 34% (of total food energy on ER) at 12:00 (chicken and salad) and 66% at 19:00 (pasta, chicken and Bolognese sauce), with a bolus of water consumed at 08:00 on ER, isovolume to the morning meal provided at 08:00 during EB. Additional water intake on both trials was prescribed at 35 mL kg^−1^ body mass [2516 (283) mL] and was evenly distributed throughout the day. Diets were tailored to participants’ preferences. The ER diet was created by primarily removing high-carbohydrate and high-fat foods from the EB diet to preserve protein content and ensure similar foods were provided on day 1 for both trials. Lean body mass (LBM) was derived from body mass and body fat percentages determined from skinfold measurements collected at baseline [23]. It was estimated that the EB diet provided 40 (2) kcal kg LBM^−1^ and the ER diet provided 10 (1) kcal kg LBM^−1^. Participants were asked to perform minimal physical activity when outside the laboratory, including the strict avoidance of any structured exercise.

**Fig. 1:**
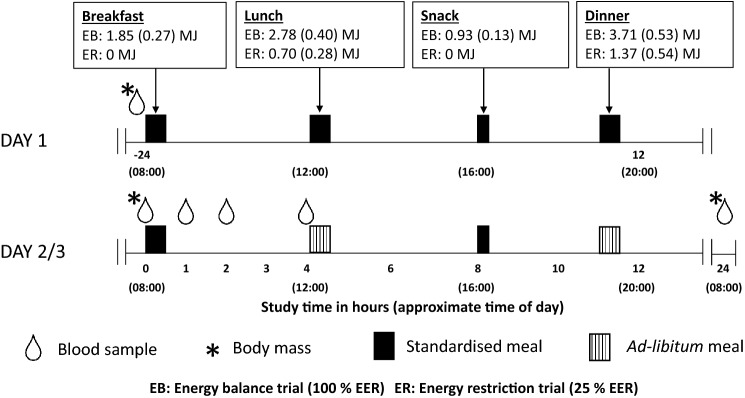
Schematic representation of study design

On day 2, participants arrived at 07:30, and remained in the laboratory until 20:00, with all food consumed in the laboratory. A fasted blood sample was collected (0 h; 08:00) from an indwelling cannula, after which participants consumed a standardised breakfast consisting of cereal, semi-skimmed milk, white bread, jam and butter, providing 25% of EER [2.44 (0.35) MJ; 93 (14) g carbohydrate; 16 (2) g protein; 15(2) g fat, 3 (0) g fibre]. Further blood samples were collected 1, 2, and 4 h after commencement of this meal. Participants were provided ad libitum lunch (12:00; 4 h) and dinner (19:00; 11 h) meals, as well as a standardised mid-afternoon snack [16:00; 8 h; 0.86 (0.12) MJ], as described previously [17]. Participants left the laboratory after dinner 20:00 (12 h), but were not permitted to consume any additional food or drink (other than plain water). Participants returned to the laboratory at 07:30, the following morning (day 3) and a final blood sample was collected via venepuncture (08:00; 24 h; Fig. 1).

### Blood sample collection and analysis

C-terminal telopeptide of type I collagen (CTX) and procollagen type 1 N-terminal propeptide (P1NP) were selected as markers of bone resorption and formation, as recommended by the International Osteoporosis Foundation and the International Federation of Clinical Chemistry [24]. Parathyroid hormone (PTH) was also measured as a marker of calcium metabolism. For each sample, blood was collected from an antecubital vein after at least 30 min of supine rest, and was dispensed into pre-chilled tubes containing EDTA (1.75 mg mL^−1^). Plasma was separated by centrifuge (15 min; 1750*g*; 4 °C) and the supernatant was stored at − 20 °C for 24 h, before being transferred to − 80 °C for later analysis. Samples collected at − 24, 0, 1, 2, 4 and 24 h were analysed for concentrations of total P1NP (Bioassay Technology Laboratory, Shanghai, China; CV ≤ 12.5%), CTX (Immunodiagnostic Systems, West Bolden, UK; CV ≤ 5.2%) and PTH (DiaMetra, Milan, Italy; CV ≤ 10.3%) by ELISA.

### Statistical analysis

Data were analysed using SPSS 24.0 (SPSS Inc, Chicago, USA). All data were checked for normality using a Shapiro–Wilk test. Repeated measures ANOVA was used to evaluate main effects of time, trial and time-by-trial interactions, followed where necessary, by Holm–Bonferroni adjusted post hoc paired *t* tests (normally distributed data) or Wilcoxon signed-ranks tests (non-normally distributed data). Total area under the curve (AUC) was calculated in response to the standard breakfast (0–4 h) using the trapezoidal method and were analysed using a *t* test or Wilcoxon signed-ranks test, as appropriate. Sex was also entered as a between-participant factor in repeated measures to test for sex-by-time-by-trial and sex-by-trial interaction effects. Data sets were determined to be significantly different when *P* < 0.05. Data are presented as mean (1SD) unless stated otherwise.

## Results

### Body mass

There were main sex-by time (*P* < 0.05) and sex-by-time-by-trial (*P* < 0.05) effects, whereby body mass loss during ER between day 1 and day 2 was greater in males than females (*P* < 0.001). However, these differences were due to the larger body mass of males compared to females, as when presented as a percentage change in body mass, no differences were noted (*P* = 0.524) and, therefore, male and female data are presented together. There were time (*P* < 0.001) and interaction (*P* < 0.001) effects for body mass. Body mass was not different between trials on day 1 (*P* = 0.059) or day 3 (*P* > 1.00), but was lower during ER on day 2 (*P* < 0.01). Body mass decreased from day 1 to day 2 during both trials, but to a greater extent during ER (*P* < 0.001; Table 2).

**Table 2:** Morning body mass measurements during each day of each experimental trial

	Energy balance (EB)			Energy restriction (ER)		
	Day 1 (− 24 h)	Day 2 (0 h)	Day 3 (24 h)	Day 1 (− 24 h)	Day 2 (0 h)	Day 3 (24 h)
Body mass (kg)	69.2 (9.6)	68.9 (9.6)*	68.9 (9.6)*	69.5 (9.8)	68.4 (9.5)^†,^*	68.9 (9.7)*
Δ from day 1 (kg)	–	− 0.3 (0.3)	− 0.3 (0.5)	–	− 1.1 (0.4)^†^	− 0.6 (0.4)^†^

Values are mean (1SD)

*EB* energy balance trial, *ER* energy restriction trial

^†^Significant difference to EB at corresponding time point

*Significant difference to − 24 h during the same trial (*P* < 0.05)

### CTX and P1NP

There were no sex-by-time-by-trial effects for CTX (*P* = 0.188) or P1NP (*P* = 0.426), so male and female data are presented together. There were no differences between trials at − 24 h (baseline) in plasma CTX (*P* = 0.532) or P1NP (*P* = 0.548) concentrations.

**Fig. 2:**
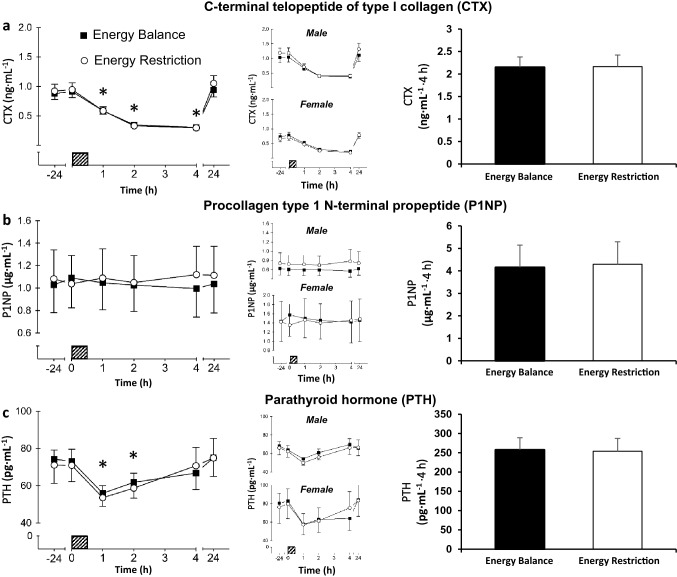
Top panel shows C-terminal telopeptide of type I collagen (CTX) (**a**), middle panel shows procollagen N-terminal propeptide (P1NP) (**b**) and bottom panel shows parathyroid hormone (PTH) (**c**). From left to right, concentrations at each time-point for all participants (*n* = 16), concentrations at each time-point for male (*n* = 8) and female (*n* = 8) participants, total area under the curve for all participants after consuming the standardised breakfast (0–4 h; 08:00–12:00). Hatched box indicates when breakfast was consumed. Data are mean with error bars representing standard error of the mean. Asterisk indicates the value is significantly different to baseline (− 24 h)

There was a time (*P* < 0.001), but no trial (*P* = 0.489) or interaction (*P* = 0.157) effects for CTX. Compared to baseline, plasma CTX concentrations were not different at 0 h (*P* = 0.512). Plasma CTX concentrations decreased after consuming breakfast, and were lower than baseline between 1 and 4 h, (*P* < 0.001) but were not different at 24 h (*P* = 0.110). There was no difference in postprandial AUC between trials for CTX (*P* = 0.916; Fig. 2a).

There were no time (*P* = 0.773), trial (*P* = 0.584) or interaction (*P* = 0.148) effects for P1NP (Fig. 2b). There was no difference in postprandial AUC between trials for P1NP (*P* = 0.661; Fig. 2b).

### PTH

There were no sex-by-time-by-trial (*P* = 0.471) effects for PTH, so male and female data are presented together. There were no differences between trials in fasting plasma PTH (*P* = 0.573) concentrations at − 24 h (baseline).

There was a time effect (*P* < 0.001), but no trial (*P* = 0.566) or interaction (*P* = 0.575) effects for PTH. Compared to baseline, plasma PTH concentrations were similar at 0 h (*P* = 1.00). Plasma PTH concentrations decreased after consuming breakfast and were lower than baseline at 1 h (*P* < 0.001) and 2 h (*P* < 0.05), but not 4 h or 24 h (*P* = 1.00). There was no difference in postprandial AUC between trials for PTH (*P* = 0.678; Fig. 2c).

### Energy intake

There was a between-participant main effect of sex, with ad libitum energy intake greater in males compared to females (*P* < 0.001), but there were no sex-by-trial (*P* = 0.614) or sex-by-time-by-trial (*P* = 0.086) interaction effects for energy intake, so male and female data are presented together.

Energy provided in the pre-prepared diets on day 1 was 6.94 (1.00) MJ lower on ER compared to EB (Table 2). On day 2, total ad libitum energy intake (i.e. lunch and dinner) was greater during ER compared to EB [EB: 8.62 (2.14) MJ; ER: 9.33 (2.14) MJ; *P* < 0.05]. The greater energy intake on day 2 [0.71 (1.27) MJ] was sufficient to replace ~ 11% of the energy deficit created on day 1 (Table 3).

**Table 3:** Total and relative energy and macronutrient intake during each day of the experimental trial

	Day 1		Day 2		Average	
	EB	ER	EB	ER	EB	ER
Protein (g)	97 (15)	60 (9)^†^	93 (20)	97 (18)	95 (16)	78 (12)^†^
Protein (g kg BM^−1^)	1.39 (0.11)	0.87 (0.08)^†^	1.34 (0.26)	1.41 (0.21)	1.37 (0.17)	1.14 (0.13)^†^
Carbohydrate (g)	293 (44)	56 (8)^†^	402 (91)	419 (98)	347 (64)	237 (52)^†^
Carbohydrate (g kg BM^−1^)	4.21 (0.11)	0.81 (0.05)^†^	5.77 (1.11)	6.09 (1.19)	4.99 (0.70)	3.45 (0.61)^†^
Fat (g)	69 (10)	9 (1)^†^	90 (23)	99 (21)^†^	80 (15)	54 (11)^†^
Fat (g kg BM^−1^)	0.99 (0.06)	0.13 (0.01)^†^	1.30 (0.06)	1.46 (0.30)^†^	1.14 (0.16)	0.79 (0.15)^†^
Fibre (g)	11 (2)	3 (1)^†^	21 (5)	22 (5)	16 (3)	13 (3)^†^
Fibre (g kg BM^−1^)	0.16 (0.01)	0.05 (0.00)^†^	0.31 (0.06)	0.32 (0.06)	0.23 (0.04)	0.18 (0.03)^†^
Energy (MJ)	9.23 (1.34)	2.33 (0.34)^†^	11.91 (2.49)	12.62 (2.46)^†^	10.59 (1.83)	7.48 (1.36)^†^
Energy (kJ kg BM^−1^)	133 (9)	34 (2)^†^	171 (30)	184 (29)^†^	157 (19)	109 (15)^†^

Values are mean (1SD) and are presented in grams (g), grams per kilogram of body mass (g kg BM^−1^), megajoules (MJ) and kilojoules per kilogram of body mass (kJ kg BM^−1^)

*EB* energy balance trial, *ER* energy restriction trial

^†^Significantly different from EB (*P* < 0.05)

## Discussion

The present study shows that an acute episode of severe energy restriction has no effect on markers of bone metabolism in the fasted state, or for 4 h after consuming a high-carbohydrate breakfast. Although short periods of severe energy restriction (such as intermittent fasting) might not, therefore, have a deleterious effect on bone health, longer term studies utilising radiological scanning to elicit a more direct assessment of bone health are warranted.

This study assessed the effect of an acute 24 h period of severe energy restriction on markers of bone formation and resorption in fasted and postprandial states, finding that a diet providing ~ 10 kcal kg LBM^−1^ had no immediate effects on markers of bone metabolism. In the fasted state, studies of longer periods of continuous energy restriction have observed a dose–response decrease in markers of bone formation (P1CP) when energy availability was restricted to 10, 20 or 30 kcal kg LBM^−1^ day^−1^ for 5 days, but N-terminal telopeptide (NTX; a marker of bone resorption) was only increased in the 10 kcal kg LBM^−1^ day^−1^ condition [12]. Similarly, Papageorgiou et al*.* [11] showed a decrease in a marker of bone formation (P1NP), but no change in a marker of bone resorption (CTX) after restricting energy availability to 15 kcal kg LBM^−1^ day^−1^ for 3 days. In combination with the current study, these studies suggest that the length of energy restriction or the total energy deficit created is important when considering the effect on bone formation and resorption. It may also be that a threshold in duration or magnitude exists, or a combination of these two factors, before energy restriction elicits a deleterious effect on bone metabolism, although this remains to be systematically investigated.

The current study also found there was no effect on markers of bone metabolism to feeding the following day. This is important as feeding is critical to the bone remodelling process [8]. As the current study only assessed the postprandial response to the first meal consumed after energy restriction, it is possible that delayed changes occurring in the subsequent hours or days were not captured. Studies that assess bone metabolism in response to multiple meals over multiple days would be required to elucidate this. The current data indicate that diets involving short periods of severe energy restriction alternated with longer periods of adequate energy intake might not detrimentally affect bone health. Whilst further research is required to determine the chronic effects, intermittent energy restriction diets may aid bone health to a greater extent than continuous energy restriction diets, which have consistently been shown to reduce bone health [25].

Consistent with the results of the current study, Barnosky et al*.* [13] found markers of bone metabolism in the fasted state were unchanged after 6-month alternate-day modified fasting or continuous energy restriction. Although surprisingly, Barnosky et al*.* [13] also reported no change in bone mineral content or BMD, in any condition, despite participants losing approximately 8% body mass. These measurements of bone remodelling were a secondary analysis of a larger study [26], which also found that when participants were permitted to self-select their diet, the disparity between ‘fasting’ and ‘fed’ days was less than 500 kcal by study end-point. It is, therefore, debatable whether this study truly assessed the effects of intermittent severe energy restriction dieting. Nevertheless, weight-loss achieved via continuous energy restriction has been consistently shown to lead to reduced BMD [25]. Issues with dual-energy X-ray absorptiometry (DXA) in participants losing weight [8, 27], as well as difficulty controlling exercise [28] and diet composition (e.g. calcium intake) [29], over a 6-month intervention period involving 3-months of self-selected food intake, might help to explain why these results differ from other diet-induced weight-loss studies.

The present study is the first to report how bone metabolic markers respond after energy restriction in the postprandial state, finding that 24 h of severe energy restriction did not affect markers of bone resorption or bone formation in response to feeding. Consuming a meal of individual or mixed macronutrient content causes a rapid suppression of bone resorption markers [14-16]. Feeding, therefore, acutely influences markers of bone metabolism, but whether this response is affected after a dietary intervention is rarely considered. Given the importance of feeding to bone metabolism, an impaired postprandial bone metabolic response could be indicative of a deleterious effect on BMD. Consistent with most acute studies, this study showed CTX (marker of bone resorption) was strongly suppressed after feeding, with no change in P1NP (marker of bone formation). Importantly, the postprandial bone metabolic response was similar, whether participants had consumed 25% (~ 10 kcal kg LBM^−1^) or 100% (~ 40 kcal kg LBM^−1^) of their energy requirements the previous day, indicating that a single 24 h period of severe energy restriction does not interfere with the subsequent postprandial bone remodelling response. How bone metabolism responds during the energy restriction period itself remains unknown. It seems likely that energy restriction of this severity would have a negative impact on bone metabolism, but it would be interesting to explore in future studies what the net effect is of 24 h severe energy restriction and subsequent ad libitum refeeding on bone remodelling.

PTH is recognised as a key factor in bone remodelling, with elevated concentrations increasing bone turnover [30]. Results of the present study demonstrate that 24 h of severe energy restriction does not affect PTH concentrations, indicating that PTH does not change in response to acute perturbations in energy balance. Whilst no difference was shown in PTH, the original study on which this secondary analysis was performed showed that several factors associated with bone metabolism were affected by 24 h of severe energy restriction. After consuming a standardised meal, Clayton et al*.* [17] showed elevated plasma glucose, suppressed plasma acylated ghrelin and a tendency (*P* = 0.06) for elevated plasma insulin concentrations, after 24 h severe energy restriction compared to 24 h of adequate energy intake. A similar study involving a short period of severe energy restriction (< 10% EER for 48 h) with subsequent refeeding similarly showed these changes, as well as elevated peptide P, glucagon-like peptide-1 and total ghrelin [18]. Despite several of these endocrine changes being linked to bone metabolism [15], no postprandial changes in markers of bone metabolism in response to acute energy restriction were observed in the current study.

Mechanistic evidence explaining the involvement of endocrine factors on bone remodelling is derived primarily from studies that have identified corresponding receptors expressed on osteoblasts and/or osteoclasts [31]. However, in vivo evidence showing a clear effect of these endocrine factors on bone metabolism is lacking. For example, diabetes and insulin resistance are associated with increased fracture risk [32], but glucose-clamp studies infusing insulin across the physiological range showed no change in bone formation or resorption, suggesting insulin per se does not directly impact bone metabolism [33]. The results of the current study indicate that insulin and other aforementioned endocrine factors are not involved in the immediate post-prandial bone metabolic response following severe energy restriction. However, the time-course response for changes in markers of bone metabolism is not fully established. Although no differences were noted in fasting concentrations on days 1 and 2, it is possible that the observed postprandial changes in associated endocrine factors precede changes in bone metabolism. Therefore, this might not have been captured by the current study. Studies with longer monitoring periods, for example, over multiple restriction-repletion cycles and in response to several meals, are required to fully establish whether an acute episode of energy restriction affects bone remodelling. In addition, future studies should aim to investigate the cause of altered bone metabolism through dietary manipulation, by assessing fluctuations in hormones (e.g. incretin hormones and those associated with bone regulatory pathways such as Wnt signalling) that have a suggested association with bone metabolism.

Alongside diet, exercise is considered to be important for achieving successful long-term weight management [34]. Weight-bearing exercise is typically considered to have an anabolic effect on bone initiated via an increase in bone loading [35]. However, the extent to which exercise increases BMD is likely to be, at least partially, mediated by energy availability. A recent study [36] showed that restricting energy intake after a morning high-intensity exercise session to ~ 10 kcal kg BM^−1^ resulted in greater concentrations of CTX in comparison to consuming a diet providing ~ 52 kcal kg BM^−1^. This study also showed that energy provided specifically by carbohydrate, rather than overall energy availability was important in attenuating bone resorption after exercise. These results suggest the undertaking of exercise during intermittent energy restriction, particularly when implemented with carbohydrate restriction, may have a detrimental effect on bone metabolism.

Traditionally, intermittent fasting has been considered a means of achieving weight loss rather than weight maintenance. It is unclear from the current study whether acute severe energy restriction would prompt a similar bone metabolic response in an overweight or obese population. Due to relatively greater bone loading caused by carrying additional weight, it might be expected that bone turnover would be increased in obese individuals, causing the accrual of bone mass if the loading stimulus is of an appropriate magnitude [25]. In line with Hammond et al*.* [36], it could be speculated that reduced energy and/or carbohydrate intake in this population may impair optimal bone remodelling leading to an increase in bone resorption. Future studies should aim to investigate the effects of severe energy restriction in overweight and obese individuals, as well as people at increased risk of fragility fracture, such as post-menopausal women.

In conclusion, 24 h of severe energy restriction had no effect on markers of bone resorption or bone formation. The acute nature of the intervention may explain why bone metabolism was unchanged. This suggests that short-term severe energy restriction as a method of weight control might not negatively affect bone health, but further studies are required to determine how repeated episodes to severe energy restriction influence long-term bone accrual.

## Electronic supplementary material

Below is the link to the electronic supplementary material.

Supplementary file1 (XLSX 71 kb)
